# Utility of Integrated PET/MRI for the Primary Diagnostic Work-Up of Patients with Ewing Sarcoma: Preliminary Results

**DOI:** 10.3390/diagnostics12102278

**Published:** 2022-09-21

**Authors:** Michal Chodyla, Francesco Barbato, Uta Dirksen, Julian Kirchner, Benedikt M. Schaarschmidt, Bernd Schweiger, Michael Forsting, Ken Herrmann, Lale Umutlu, Johannes Grueneisen

**Affiliations:** 1 Department of Diagnostic and Interventional Radiology and Neuroradiology, University Hospital Essen, University of Duisburg-Essen, D-45147 Essen, Germany; 2 Clinic of Nuclear Medicine, University Hospital Essen, University of Duisburg-Essen, D-45147 Essen, Germany; 3 Clinic for Pediatrics III, University Hospital Essen, University of Duisburg-Essen, D-45147 Essen, Germany; 4 Department of Diagnostic and Interventional Radiology, Medical Faculty, University of Dusseldorf, D-40225 Dusseldorf, Germany

**Keywords:** PET/MRI, Ewing sarcoma, staging, treatment monitoring

## Abstract

Background: This study was conducted to evaluate the clinical applicability of integrated PET/MRI for staging and monitoring the effectiveness of neoadjuvant chemotherapy in Ewing sarcoma patients. Methods: A total of 11 juvenile patients with confirmed Ewing sarcoma, scheduled for induction polychemotherapy, were prospectively enrolled for a PET/MR examination before, during and after the end of treatment. Two experienced physicians analysed the imaging datasets. They were asked to perform a whole-body staging in all three examinations and to define treatment response according to the RECIST1.1 and PERCIST criteria for each patient. Results: In eight patients lymph node and/or distant metastases were detected at initial diagnosis. According to the reference standard, three patients achieved complete response, six patients partial response, and one patient showed stable disease while another patient showed progressive disease. RECIST1.1 categorized the response to treatment in 5/11 patients correctly and showed a tendency to underestimate the response to treatment in the remaining six patients. PERCIST defined response to treatment in 9/11 patients correctly and misclassified two patients with a PR as CR. Conclusion: PET/MRI may serve as a valuable imaging tool for primary staging and response assessment of juvenile patients with Ewing sarcoma to induction chemotherapy, accompanied by a reasonable radiation dose for the patient.

## 1. Introduction

Ewing sarcoma is the second most common malignant bone tumor of pediatric and juvenile patients [1]. An accurate initial diagnostic of these tumors is of particular importance to select the most appropriate multimodal therapeutic concept, frequently starting with induction polychemotherapy according to the VIDE-regimen, followed by local treatment of the primary tumor [2]. In addition, reliable information about metastatic spread is of high importance to plan further therapeutic steps and to assess the patient’s prognosis. Based on its higher soft-tissue contrast compared to other morphological imaging techniques, MRI is considered the imaging modality of choice for regional tumor evaluation [3]. A precise determination of the primary tumor extant is of high importance for the selection of the local treatment procedure. Moreover, hybrid imaging with the use of 18F-FDG PET has been shown to be an efficacious imaging technique for staging and restaging malignant bone tumors. In this context, previous studies reported that PET/CT enables the identification of lymph node or bone metastases with a higher sensitivity than conventional imaging modalities [4,5,6]. In addition, 18F-FDG PET provides valuable information about tumor metabolism that can be used for the discrimination of viable or nonviable tumors as well as to assess the effectiveness of therapeutic interventions due to measurable changes of the FDG-uptake under treatment [7].

The implementation of PET/MRI scanners enables the simultaneous acquisition of complementary metabolic and MR-derived morphologic and functional imaging datasets and therefore arises the potential for a characterization and staging of primary bone tumors within a single imaging session [8,9,10]. Especially, for the frequently young patient population with Ewing sarcomas, these integrated imaging systems could be of value, as they provide a notable reduction in ionizing radiation when compared to PET/CT scanners due to the omission of the CT-component [11,12]. Therefore, this preliminary study was initiated to assess the clinical applicability and diagnostic value of PET/MR imaging for the initial staging and monitoring of the effect of neoadjuvant chemotherapy in patients with primary Ewing sarcoma.

## 2. Material and Methods

### 2.1. Patients

The present study was approved by the institutional review board and written informed consent was obtained from all patients before each examination. A total of 11 juvenile patients (median (interquartile range, IQR) 17 (3) years; range 15–25 years) with the histopathological confirmation of an Ewing sarcoma and scheduled for 6 cycles of the VIDE polychemotherapy regimen (vincristine, ifosfamide, doxorubicin and etoposide) were enrolled in this study. All patients underwent a 18F-FDG PET/MRI examination for primary whole-body tumor staging. In addition, further 18F-FDG PET/MRI scans were performed under therapy as well as upon completion of induction chemotherapy to evaluate the effectiveness of neoadjuvant treatment.

### 2.2. PET/MR Imaging

PET/MRI scans were performed on a 3 Tesla integrated PET/MR system (Biograph mMR, Siemens Healthineers, Germany) and with a delay of 60 min after a body weight-adapted dosage of *18F-fluorodeoxyglucose* (18F-*FDG*) was injected intravenously. Calculated activity of administered tracer agent amounted to [median (IQR)] 212 (46) MBq (1st scan), 198 (39.8) MBq (2nd scan) and 191 (23.8) (3rd scan), respectively. Whole-body PET/MR imaging data were obtained in 4–5 bed positions (depending on patients’ size) with a PET-data acquisition time of 4 min per bed. In addition, PET-data of the primary tumor were obtained with an acquisition time of 15 min. PET images were reconstructed subsequently, using the iterative ordered-subset expectation maximization algorithm, 3 iterations and 21 subsets, a Gaussian filter with 4.0 mm full width at half maximum and a 344 × 344 image matrix. For attenuation correction, a vendor supplied software solution with a four-compartment-model attenuation map (μ-map) calculated from fat-only and water-only datasets, as obtained by Dixon-based sequences was used. MR imaging datasets were acquired simultaneously to PET-data. MR sequence protocols which were used, in dependence of the localization of the primary tumor, are displayed within the (Supplements Tables S1–S3).

### 2.3. Image Analysis

Readings of PET/MR imaging datasets were performed by two physicians with 7 and 8 years of experience in reading MRI and hybrid imaging, using a dedicated viewing software for hybrid imaging (Syngo.via; Siemens, Healthcare, Erlangen, Germany). Both readers were informed about the diagnosis of a primary manifestation of a Ewing sarcoma but were blinded to the patients’ identification data, the outcome of induction chemotherapy and subsequent therapeutic interventions. In a first session, they were instructed to perform a whole-body tumor staging for each patient. They were asked to identify the primary tumor manifestation as well as to detect metastatic spread. In cases of a metastatic dissemination, the total number of metastases, which were included in the rating was limited to ten, with a maximum of two lesions per organ. For size measurements of primary Ewing sarcomas that arose in the bone, only the soft-tissue component was considered, and the maximum tumor size was determined in only one dimension. In addition, for all tumor lesions the standardized uptake value was measured. In a second reading session, interim scans of all patients and in a third session the examinations at the end of treatment were analyzed. Therefore, the readers were instructed to evaluate potential changes of the initially detected lesions (by measurements of the tumor size and metabolic activity) as well as to identify the occurrence of new tumor lesions.

Treatment response was evaluated according to the RECIST 1.1 and PERCIST criteria [13,14]. Therefore, each patient was assigned to one of the following categories: partial response, complete response, stable disease, or progressive disease, based on the findings of the staging and follow-up examinations.

### 2.4. Reference Standard

In each case, the diagnosis of Ewing sarcoma was confirmed by histology after biopsy. In seven patients, primary tumors were resected after completion of induction chemotherapy and the results from histology were used to determine the amount of vital tumor. In the remaining patients, who underwent further radiation therapy or consolidation chemotherapy, follow-up imaging with a minimum time period of one year, [median (IQR), 30 (19.5) months] comprising at least one hybrid imaging examination (Table 1), was used to determine treatment response. Furthermore, all patients underwent a chest CT examination at initial diagnosis. In addition, results from histology after biopsy or resection as well as findings in follow-up examinations were applied for the determination of metastatic spread.

## 3. Results

All primary PET/MR scans as well as subsequent staging examinations were successfully completed without any relevant side effects. The effective dose of the administered 18F-FDG amounted to [median (IQR)] 4 (0.9) mSv (1st scan), 3.8 (0.7) mSv (2nd scan) and 3.6 (0.5) mSv (3rd scan), respectively. In seven of the 11 patients the primary tumor arose in the bone and in the remaining 4 patients in the soft tissue. Furthermore, in eight patients lymph node and/or distant metastases were present at initial diagnosis, and PET/MRI could correctly identify the occurrence of metastatic spread in all eight cases. In three of these eight patients, lung metastases were present and PET/MRI enabled correct identification in two of the three cases, whereas in one patient metastatic spread to the lungs was not detected in the primary staging examination. Patients’ characteristics, comprising information about the primary tumor site and presence of metastases are displayed in Table 2.

**Figure 1:**
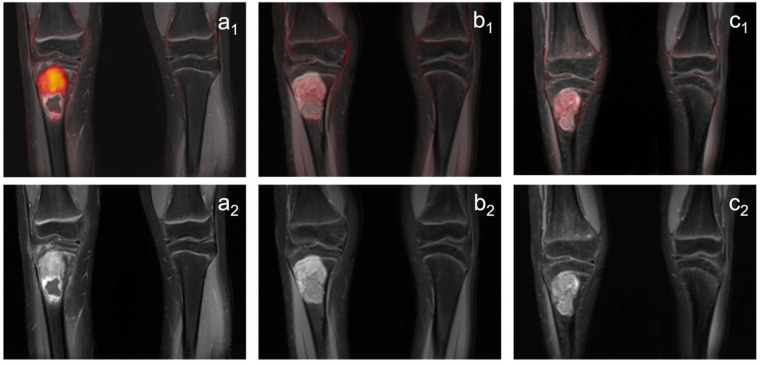
Images of a patient with a Ewing sarcoma in the proximal right tibia. The tumor shows a pathologic glucose metabolism (SUVpeak: 7.2) and a maximum diameter of 64 mm at the pretherapeutic scan: ((**a_1_**): PET/MRI; (**a_2_**) MRI). The metabolic activity decreases significantly under treatment (PET/MRI; (**b_1_**): first follow-up, SUVpeak: 1,9 (−73.6%); (**c_1_**): second follow-up, SUVpeak: 1,7 (−76.4%)), but the tumor reveals only a slight decrease in size (MRI; (**b_2_**): first follow-up, 57 mm (−10.9%); (**c_2_**): second follow-up, 53 mm (−17.2%)). Histopathological analysis after tumor resection did not reveal vital tumor (reference standard: complete response).

Moreover, based on the reference standard, a total of three patients revealed a complete response, six patients a partial response, one patient showed stable disease and another patient showed progressive disease. For response assessment according to the RECIST 1.1 criteria, target lesions and non-target lesions were identified and in addition, the potential occurrence of new tumor lesions was evaluated. Mean sum of the longest diameters at the baseline examinations was 97.2 ± 35.2 mm, [median (IQR)] 71 (13) mm and showed a slight decrease to 85.4 ± 28.1 mm (−12.1%), 62 (17) mm at the first follow-up scan and a moderate decrease to 68.1 ± 26.9 mm (−29.9%), 53 (22) mm at the second follow-up scan. Based on the RECIST 1.1 criteria, a total of five patients showed a partial response and five patients showed stable disease (Table 2). In one case, two new bone metastases could be detected between the first and second follow-up scan, consequently the patient was categorized as progressive disease. In five of the 11 patients, treatment response was categorized correctly, in accordance with the reference standard. Among the remaining six patients, in three cases a partial response was falsely categorized as stable disease, and three further patients with a complete response were misclassified as stable disease (*n* = 2) or partial response (Figure 1).

**Figure 2:**
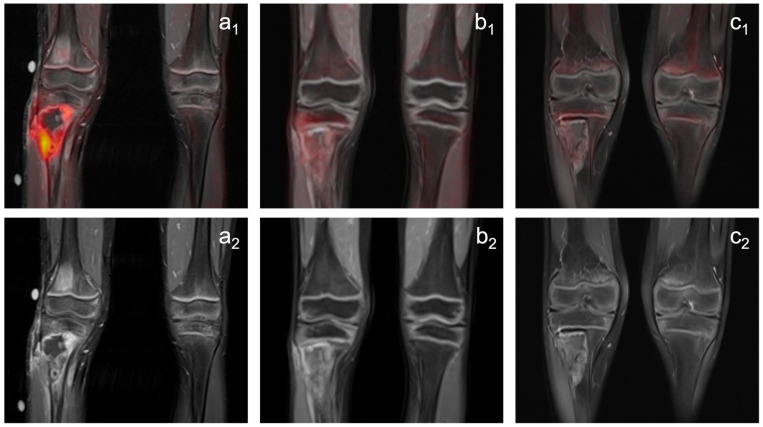
Images of a patient with a Ewing sarcoma in the proximal right tibia (74 mm), which shows a pathological tumor metabolism (SUVpeak: 4,1) at the pretherapeutic examination ((**a_1_**): PET/MRI, (**a_2_**) MRI). The tumor reveals a significant decrease in metabolic activity under treatment (PET/MRI; (**b_1_**): first follow-up, SUVpeak: 1,5 (−63.4%); (**c_1_**): second follow-up, SUVpeak: 1,2 (−70.7%)), combined with a moderate decrease in size (MRI; (**b_2_**): first follow-up, 51 mm (−31,1%); (**c_2_**): second follow-up, 41 mm (−44.6%)). Histopathological analysis after subsequent tumor resection revealed remaining vital tumor (reference standard: partial response).

Furthermore, the mean SUVpeak at the baseline examination of all patients amounted to 6.6 ± 2.5, [median (IQR)] 5.6 (1.9) and decreased to 3.6 ± 2.3 (−45.5%), 2.7 (4.1) at the first scan under treatment and to 2.8 ± 1.9 (−57.6%), 2.2 (1.3) at the second follow-up examination. According to the PERCIST criteria, five patients were categorized as a complete response, four patients as a partial response, whereas one patient showed a stable disease, and one further patient was classified as progressive, due to the occurrence of new metastases under treatment. In concordance with the reference standard, treatment response was correctly defined in nine cases, whereas two patients with a partial response were misclassified with a metabolic complete response at the end of treatment (Table 3, Figure 2). 

## 4. Discussion

Accurate initial diagnostics of patients with confirmation of a primary Ewing sarcoma is of particular importance to plan the most appropriate treatment strategy and to assess patients’ prognosis. The multimodal therapeutic concept usually includes induction chemotherapy followed by local treatment (e.g., surgery or radiation therapy), depending on the localization and the extent of the primary tumor manifestation [2,15].

MRI has been shown to be more accurate than other morphological imaging techniques (e.g., CT or ultrasound) for the delineation of tumor margins and the determination of tumor invasion into the adjacent anatomical structures [3]. Therefore, MR imaging is recommended in the guidelines for the evaluation of the local extent of the primary tumor as well as the identification of potential skip lesions and consequently, has high impact on surgical planning [15]. Furthermore, CT and skeletal scintigraphy are frequently used in clinical practice for the evaluation of metastatic spread. However, viable bone tumors have been shown to be FDG-avid and therefore, hybrid imaging techniques like PET/CT have been shown highly sensitive for tumor detection and are increasingly used for primary staging and the selection of appropriate biopsy sites [6,16].

Around a decade ago, integrated PET/MR scanners became available for clinical use, combining the acquisition of PET- and MR-imaging data in one examination [8]. Especially, for the assessment of bone malignancies, this imaging technique enables primary tumor characterization as well as the identification of metastatic disease within a single imaging session. The information about the tumor metabolism can additionally be applied for monitoring treatment effects. In this context, previous studies have shown that histopathological response of sarcomas can be predicted, based on changes of the metabolic activity of these tumors under therapy [17,18,19,20]. The present study evaluated the potential of integrated PET/MR imaging for staging and treatment monitoring of patients with a primary Ewing sarcoma, that underwent induction polychemotherapy according to the VIDE-regimen. In our patient cohort, morphological-based response assessment according to the RECIST criteria, did not allow for reliable determination of treatment response. In only 5 of the 11 patients, treatment effects were correctly categorized. In the remaining cases, RECIST revealed a tendency to underestimate the treatment effect, since 5 patients with a complete response or partial response were rated as stable disease and one patient with complete response was falsely classified as partial response. The phenomenon, that morphologic response criteria (e.g., RECIST) do not provide a reliable characterization of the therapeutic effect of soft tissue or bone sarcomas has been reported in previous publications [21,22]. Frequently, these tumors show structural changes under therapy, such as the development of necrosis, fibrosis, or granulation tissue and, more rarely, significant changes in tumor size [23].

Moreover, numerous trials already demonstrated the high performance of 18F-FDG PET for staging and treatment monitoring of several different tumors, including sarcomas [24,25]. Especially, the information about the metabolic activity of the primary tumor manifestation and its changes under therapy has been shown to be a more reliable parameter for the discrimination between treatment response or non-response in sarcoma patients than morphological-based tumor evaluation [26,27,28]. In concordance with these findings, response to primary systemic therapy could be correctly determined in nine of the 11 patients in our study, using the PERCIST criteria. In the two other cases, treatment effects were over-estimated, and the patients were misclassified as complete response instead of a partial response. Histopathological analysis also revealed a good response in these cases but identified a remaining amount of vital tumor parts of almost 10%. Nevertheless, FDG-PET appears to be a reliable tool for monitoring neoadjuvant systemic therapy of Ewing sarcoma patients.

However, a well-known drawback of integrated PET/MR imaging is the identification and characterization of lung nodules, particularly due to limitations of the MR-component in lung imaging. Previous comparative studies could already demonstrate that the detection rate of PET/MRI remains inferior to that of PET/CT, especially because of its lower ability to detect pulmonary lesions smaller than 10 mm [29,30]. This is also reflected by the results of our study, in which pulmonary metastases could be identified in only two of three patients. Therefore, when PET/MRI is applied for primary staging, we recommend an additional low-dose thoracic CT scan, in order not to miss a potential metastatic spread to the lungs, which represents one of the most common metastatic sites of sarcomas. On the other hand, this examination implies an additional radiation exposure for the usually juvenile patients, which adds to the ionizing radiation dose of the PET/MR scan, caused by the FDG-PET component. However, combining these examinations provides a highly accurate primary tumor evaluation and a high sensitivity for the identification of metastatic spread. This valuable information can be used for biopsy and treatment planning of patients with Ewing sarcoma, accompanied with an overall moderate and appropriate radiation exposure for the patient, when compared to the conventional staging algorithm.

The present study is not without limitations. Due to the relatively small number of patients, our results should be considered as preliminary and need to be confirmed in future studies with larger patient cohorts. Moreover, we could not obtain histopathological samples of all tumors after completion of induction chemotherapy since in some cases patients underwent radiation therapy as local treatment or further systemic therapy before surgical treatment. In these cases, patients were only included when follow-up imaging with a time period of at least one year was available, comprising at least one hybrid imaging examination, in order to determine the response to therapy.

## 5. Conclusions

These preliminary results show a good performance of integrated PET/MRI for the diagnostic work-up of patients with a primary Ewing sarcoma, combining the strength of the MR-component for local staging of the primary tumor and FDG-PET for the identification of metastatic spread. In addition, FDG-PET provides valuable information about the metabolism activity of the tumor and seems to be a valuable tool for monitoring treatment effects of Ewing sarcomas. Therefore, considering the reasonable radiation exposure for the mostly young patient cohorts, even combined with a low-dose chest CT for more reliable lung assessment, integrated PET/MRI seems to be a promising imaging modality for the evaluation of patients with Ewing sarcoma.

## Figures and Tables

**Table 1:** Follow-up of the patient cohort: imaging modalities and number of scans, time period of imaging follow-up, localization of tumor progression/tumor relapse, disease-free survival (DFS).

Patient	Imaging Modality				Follow-Up (Months)	Tumor Progression	DFS (Months)
	PET/MRI	PET/CT	MRI	CT			
1	8	1	2	8	46	Bone, Peritoneum	0
2	2	-	-	2	13	Bone	6
3	8	-	5	7	45	-	-
4	5	-	6	6	28	-	-
5	5	-	9	18	41	Lung	31
6	2	1	7	8	30	Lung, Peritoneum	14
7	3	-	7	6	33	Lung, Lymph nodes	28
8	5	-	4	10	34	-	-
9	2	-	3	3	18	-	-
10	2	-	4	6	18	-	-
11	4	-	3	4	17	Bone	12

**Table 2:** Patients’ characteristics.

Patient	Sex	Age	Primary Tumor Localization (b: Bone, s: Soft-Tissue)	Metastases	Treatment after Induction Chemotherapy
1	m	19	Right scapula (b)	Bone, Soft-tissue	Chemotherapy (VAI)
2	m	23	Left iliac bone (b)	Bone, Lung	Chemotherapy (VAI)
3	m	15	Right tibia (b)	Bone, Soft-tissue	Surgery
4	f	15	Right tibia (b)	-	Surgery
5	m	25	Left pubic bone (b)	Lymph node, Lung, Soft-tissue	Radiation therapy
6	m	18	Pelvis (s)	-	Surgery
7	f	18	Pelvis (s)	Lymph node	Surgery
8	f	17	Palm of right hand (s)	-	Radiation therapy
9	m	16	Left chest wall/Rib (b)	Lymph node	Surgery
10	m	15	Right gluteal muscle (s)	Lung, Soft-tissue	Surgery
11	m	16	Left acetabulum (b)	Bone	Chemotherapy (VAI)

**Table 3:** Results of the ratings according to the RECIST 1.1- and PERCIST-criteria (CR: complete response, PR: partial response, SD: stable disease, PD: progressive disease).

Patients	Reference Standard	RECIST	PERCIST
1	PD	PD	PD
2	SD	SD	SD
3	PR	SD	CR
4	CR	SD	CR
5	PR	PR	PR
6	PR	PR	CR
7	CR	SD	CR
8	PR	PR	PR
9	CR	PR	CR
10	PR	SD	PR
11	PR	SD	PR
Correct ratings	11	5	9

## Data Availability

Available upon reasonable request.

## Supplementary Materials

The following supporting information can be downloaded at: https://www.mdpi.com/article/10.3390/diagnostics12102278/s1, Table S1: MR imaging parameters—lower extremity; Table S2: MR imaging parameters—upper extremity; Table S3: MR imaging parameters—whole-body.
