# Supplementary material for: Utility of Integrated PET/MRI for the Primary Diagnostic Work-Up of Patients with Ewing Sarcoma: Preliminary Results
Source: Diagnostics (Basel). 2022 Sep 21;12(10):2278. doi: 10.3390/diagnostics12102278 (PMC9600118; doi:10.3390/diagnostics12102278)
Supplement: Supplementary file 1 [file diagnostics-12-02278-s001.zip › diagnostics-1812298-supplementary.pdf]

**MR sequence**	**Plane**	**Slice thickness (mm)**	**Repetition time/ Echo time (ms)**	**Field of view (mm)**	**Phase FoV (%)**	**Matrix size**
T1w VIBE Dixon	coronal	3.12	3.6 / 1.23 (1st) and 2.46 (2nd)	500	65.6	192 x 79
STIR	coronal	5	5990 / 57<br>Inversion time: 220ms	380	75.0	384 × 288
T1w TSE	axial	5	616 / 12	380	68.8	512 × 256
T2w TSE	axial	5	4860 / 106	380	68.8	512 × 256
DW EPI (b-values: 0, 500, 1000 s/mm2)	axial	5	7400 / 72	420	75.0	160 × 120
T1w VIBE dynamic imaging<br>fat saturated	axial	3.5	4.32 / 2.21	380	68.8	512 × 308
T1w TSE post contrast fat saturated	coronal	5	542 / 13	380	75	512 × 256
T1w TSE post contrast fat saturated	axial	5	663 / 13	380	68.8	512 × 256

Table S1: MR imaging parameters – lower extremity.

**MR sequence**	**Plane**	**Slice thickness (mm)**	**Repetition time/ Echo time (ms)**	**Field of view (mm)**	**Phase FoV (%)**	**Matrix size**
T1w VIBE Dixon	coronal	3.12	3.6 / 1.23 (1st) and 2.46 (2nd)	500	65.6	192 x 79
STIR	coronal	5	5040 / 57 Inversion time: 220ms	380	75.0	384 × 288
T1w TSE	axial	5	650 / 12	300	75.0	448 × 256
T2w TSE	axial	5	4860 / 106	380	68.8	512 × 256
DW EPI (b-values: 0, 500, 1000 s/mm2)	axial	6	6000 / 58	380	78.0	164 × 120
T1w VIBE dynamic imaging fat saturated	axial	3.5	4.32 / 2.21	380	68.8	512 × 308
T1w TSE post contrast fat saturated	coronal	5	602 / 13	380	75	512 × 256
T1w TSE post contrast fat saturated	axial	5	722 / 13	300	68.8	448 × 256

Table S2: MR imaging parameters – upper extremity.

MR sequence	Plane	Slice thickness (mm)	Repetition time/ Echo time (ms)	Field of view (mm)	Phase FoV (%)	Matrix size
T1w VIBE Dixon	coronal	3.12	3.6 / 1.23 (1st) and 2.46 (2nd)	500	65.6	192 x 79
DW EPI (b-values: 0, 500, 1000 s/mm2)	axial	5	9900 / 82	420	75.0	160 × 90
T2w HASTE	coronal	5	1500 / 117	450	81.3	320× 211
T1w VIBE post contrast fat saturated	axial	3.5	4.08 / 1.51	400	75	448 × 230

Table S3: MR imaging parameters – whole-body.
